# Maternal Serum Isthmin-1 as a Novel Biomarker for Late-Onset Fetal Growth Restriction

**DOI:** 10.3390/jcm15187018

**Published:** 2026-09-10

**Authors:** Özge Öztürk, Recep Taha Ağaoğlu, Gunel Aliyeva, Mesut Şimşek, Kadriye Yakut Yücel

**Affiliations:** 1 Department of Perinatology, Etlik City Hospital, Ankara 06170, Turkey; tahaagaoglu@hotmail.com (R.T.A.); dr.aliyeva87@gmail.com (G.A.); yakutkadriye@hotmail.com (K.Y.Y.); 2 Department of Obstetrics and Gynecology, Etlik City Hospital, Ankara 06170, Turkey; drmesutsimsek97@gmail.com

**Keywords:** Isthmin-1, fetal growth restriction, biomarker, placental insufficiency, angiogenesis

## Abstract

**Background/Objectives:** To investigate maternal serum Isthmin-1 (ISM1) levels in pregnancies complicated by fetal growth restriction (FGR) and to evaluate its potential discriminatory performance for FGR. **Methods:** This prospective cross-sectional study included 44 pregnant women with late-onset FGR and 43 gestational age-matched healthy controls. FGR was diagnosed according to one of two criteria: (1) estimated fetal weight (EFW) below the 3rd percentile, or (2) EFW between the 3rd and 10th percentile with at least one abnormal Doppler finding (Umbilical Artery Pulsatility Index (UA-PI), Middle Cerebral Artery Pulsatility Index (MCA-PI), or Cerebroplacental Ratio (CPR). Maternal serum ISM1 concentrations were measured using enzyme-linked immunosorbent assay (ELISA). Ultrasonographic parameters, Doppler indices, and perinatal outcomes were compared between groups. Correlation and receiver operating characteristic (ROC) analyses were performed to assess diagnostic performance. **Results:** Maternal ISM1 levels were significantly lower in the FGR group compared with controls [10.63 (9.95–12.10) vs. 12.40 (10.75–13.25) ng/mL, *p* = 0.003]. ISM1 correlated negatively with FGR presence (r = −0.318, *p* = 0.003) and positively with EFW percentile (r = 0.235, *p* = 0.028). ROC analysis showed moderate diagnostic accuracy Area Under the Curve (AUC) = 0.684, 95% CI: 0.575–0.779) with 70.5% sensitivity and 72.1% specificity at a cut-off ≤11.5 ng/mL. No significant association was found between ISM1 and composite adverse perinatal outcomes. **Conclusions:** Levels of ISM1 in maternal serum were found to be significantly lower in pregnancies complicated by FGR, supporting a potential association between ISM1 and impaired fetal growth. However, its moderate diagnostic performance and lack of association with adverse perinatal outcomes indicate that ISM1 should currently be regarded as a promising exploratory biomarker rather than a standalone diagnostic or prognostic marker. Further studies are needed to validate these findings and to determine whether ISM1 provides additional value when combined with established Doppler and angiogenic markers.

## 1. Introduction

Fetal growth restriction (FGR) refers to impaired fetal growth resulting from pathological processes that prevent the fetus from reaching its genetically determined growth potential, and it remains a major cause of perinatal morbidity and mortality worldwide [1,2]. According to the 2016 Delphi consensus, late-onset FGR (≥32 weeks of gestation) is diagnosed when either the estimated fetal weight (EFW) or the abdominal circumference (AC) is below the 3rd percentile, or when EFW or AC falls between the 3rd and 10th percentiles in combination with abnormal Doppler findings, including an umbilical artery pulsatility index (UA-PI) above the 95th percentile, a middle cerebral artery pulsatility index (MCA-PI) below the 5th percentile, or a cerebroplacental ratio (CPR) below the 5th percentile [1]. A downward shift of two growth quartiles in serial estimated fetal weight measurements is also considered evidence of impaired fetal growth and supports the diagnosis of late-onset FGR. Current clinical guidelines (International Federation of Gynecology and Obstetrics (FIGO), American College of Obstetricians and Gynecologists (ACOG), International Society of Ultrasound in Obstetrics and Gynecology (ISUOG)) recommend <1 as a practical cut-off for CPR [3,4,5]. The Royal College of Obstetricians and Gynaecologists (RCOG) [6] also supports these definitions. FGR is of particular clinical importance because it is associated with an increased risk of adverse perinatal and neurodevelopmental outcomes [7].

Pregnancies complicated by fetal growth restriction (FGR) are associated with an increased risk of adverse outcomes extending from the perinatal period into later life. In accordance with the postulations of the Barker hypothesis, individuals who are subjected to the condition of FGR have been shown to possess a heightened propensity to manifest cardiovascular diseases (CVD), type 2 diabetes mellitus (T2DM), and metabolic syndrome (MetS) during their later adult years [8,9]. Consequently, the early diagnosis and risk stratification of FGR, supported by biomarkers, are critical to ensure optimal both short- and long-term prognosis.

The pathophysiology of fetal growth restriction (FGR) is widely acknowledged to be intricate and involves several mechanisms, including uteroplacental insufficiency, inadequate spiral artery remodelling, oxidative stress, inflammation, and abnormal angiogenesis [10]. In this process, decreased Placental Growth Factor (PlGF) levels and increased soluble Fms-like tyrosine kinase-1 (sFlt-1) in maternal blood are characteristic findings. The sFlt-1/PlGF ratio is a well-established biomarker, particularly in the diagnosis and prognosis of preeclampsia [11,12]. Abnormal angiogenesis and angiogenic imbalance are also implicated in the pathophysiology of FGR [10].

Isthmin-1 (ISM1) is a secreted protein containing a thrombospondin type 1 repeat (TSR1) domain and an adhesion-associated domain in MUC4 and other proteins (AMOP), with diverse biological functions [13,14]. Experimental studies have demonstrated that ISM1 can modulate endothelial cell survival and angiogenic processes through pathways involving integrin αvβ5 and glucose-regulated protein 78 (GRP78) [14,15,16,17]. In addition to its vascular effects, ISM1 has been identified as an adipokine involved in glucose uptake, metabolic regulation, and developmental signalling [14,18,19]. In pregnancy-related studies, maternal serum ISM1 concentrations have been reported to be altered in gestational diabetes mellitus, while the related Isthmin family member ISM2 has been reported to be decreased in preeclampsia [20,21].

However, there is a lack of clinical evidence regarding maternal serum ISM1 levels in pregnancies complicated by late-onset FGR.

In light of this knowledge gap and the reported biological functions of ISM1, we hypothesised that maternal serum ISM1 levels might be altered in pregnancies affected by late-onset FGR. Such an association could justify further investigation of ISM1 as a potential biomarker for FGR. Nevertheless, it is unclear whether altered ISM1 levels are related to placental dysfunction, uteroplacental perfusion, angiogenic imbalance or metabolic alterations, as these mechanisms were not investigated directly in the present study. Similarly, the potential additional diagnostic or prognostic value of combining ISM1 with established clinical, Doppler, or angiogenic markers requires evaluation in future studies.

## 2. Materials and Methods

### 2.1. Study Design and Participants

We performed a prospective cross-sectional study in the Perinatology Department of Etlik City Hospital in Ankara, Türkiye, between November 2024 and April 2025. All FGR cases included in the study were classified as late-onset FGR (≥32 weeks of gestation) according to established criteria. Although the study design was prospective in terms of recruitment and sample collection, it was cross-sectional in nature with respect to data acquisition, since each participant underwent a single evaluation between 35 and 38 weeks of gestation. Maternal body mass index (BMI) was calculated using maternal weight measured at the time of blood sampling and recorded height and therefore represented maternal BMI at the time of serum ISM1 assessment. The study included 44 pregnancies affected by late-onset fetal growth restriction (FGR) and 43 healthy gestational age-matched controls. All deliveries were performed at the same tertiary care center, ensuring a standardized approach to neonatal management across both groups.

### 2.2. Exclusion Criteria

Pregnancies complicated by hypertensive disorders, including preeclampsia and gestational hypertension, pregestational and gestational diabetes mellitus, renal or other chronic systemic diseases, multiple pregnancies and major fetal anomalies were excluded. Preeclampsia and gestational hypertension were excluded according to the diagnostic criteria of the American College of Obstetricians and Gynecologists (ACOG), and all participants included in the final cohort were normotensive, without proteinuria or antihypertensive medication use. Individual systolic and diastolic blood pressure values at the time of blood sampling were not prospectively recorded as study variables.

### 2.3. Definitions

FGR was defined according to the 2020 ISUOG guidelines. Late-onset FGR (≥32 weeks) was defined according to the Delphi criteria as an estimated fetal weight (EFW) or abdominal circumference (AC) below the 3rd percentile, or an EFW or AC between the 3rd and 10th percentiles together with abnormal Doppler parameters or a downward shift of two growth quartiles in serial EFW assessments. Composite adverse perinatal outcome (CAPO) was defined as the presence of at least one of the following: 5-min Apgar score ≤ 7, fetal distress, neonatal intensive care unit (NICU) admission, continuous positive airway pressure (CPAP) requirement, or mechanical ventilation requirement.

### 2.4. Serum Sampling and Biochemical Analysis

Peripheral venous blood was obtained from all enrolled participants. All blood samples were obtained on the same day as the ultrasonographic and Doppler assessments and under fasting conditions. Blood samples underwent centrifugation at 3000 rpm for 10 min, after which the serum fraction was stored at −80 °C pending analysis. Serum ISM1 (Human Isthmin-1 (ISM1) ELISA Kit (Shanghai Coon Koon Biotech Co., Ltd., Shanghai, China)) concentrations were quantified using a commercial enzyme-linked immunosorbent assay ELISA kit in accordance with the manufacturer’s protocol. All values were multiplied by 1/5 dilution factor and reported in ng/mL.

### 2.5. Ultrasonography and Doppler Measurements

All cases were evaluated by the same perinatologist using a Voluson S10 (GE Healthcare, Chicago, IL, USA) ultrasonography device. Fetal biometry measurements (AC, EFW) and maximum vertical pocket (MVP) were recorded. Systolic/diastolic ratio (S/D) and PI values were measured for the umbilical artery (UA) and middle cerebral artery (MCA). Cerebroplacental ratio (CPR) was calculated by the formula MCA-PI/UA-PI. Amniotic fluid volume was assessed using the maximum vertical pocket (MVP) method. To minimize measurement bias, all ultrasonographic examinations were performed by the same experienced perinatologist and laboratory analyses were conducted under standardized conditions.

### 2.6. Statistical Analysis

Within-group associations between ISM1 and continuous maternal, ultrasonographic, and perinatal variables were additionally assessed using partial correlation analyses. Partial correlations were adjusted for maternal age, body mass index, and gestational age at sampling, as appropriate. To evaluate the independent association between ISM1 levels and fetal growth restriction, Firth’s penalized logistic regression analysis was performed. This approach was selected because the relatively limited sample size may increase small-sample bias in conventional maximum-likelihood logistic regression and may also increase the risk of complete or quasi-complete separation. Firth’s penalized likelihood method reduces this bias and provides more stable parameter estimates under such conditions. Fetal growth restriction was defined as the binary outcome variable, and serum ISM1 was entered into the model as a continuous predictor. The multivariable model was adjusted for maternal age, body mass index, and gravidity, which were selected a priori based on their clinical relevance. Adjusted odds ratios (aORs) with corresponding 95% confidence intervals (CIs) were calculated. A two-sided *p*-value < 0.05 was considered statistically significant. Only participants with complete clinical and laboratory data were included in the final analysis. The linearity assumption for continuous predictors was assessed using restricted cubic spline analyses with three knots. Model discrimination was evaluated using the area under the receiver operating characteristic curve (AUC), while calibration was assessed using the calibration intercept, calibration slope, calibration plot, and Brier score.

### 2.7. Sample Size Calculation

As no previous studies have compared ISM1 levels between pregnancies complicated by fetal growth restriction (FGR) and healthy pregnancies, a pilot study was conducted including 15 pregnancies with FGR and 15 healthy controls. In this pilot analysis, median ISM1 levels were 12.5 (interquartile range [IQR], 11.55–13.75) in the healthy group and 10.40 (IQR, 10.20–11.50) in the FGR group. The difference between groups was assessed using the Mann–Whitney U test, yielding a standardized Z score of 2.22. The effect size (r) was calculated as r = Z/√*N*, resulting in r = 0.405. Subsequently, r was converted to Cohen’s d using the formula d = 2r/√(1 − r^2^), corresponding to a Cohen’s d of 0.89. Based on this effect size, an a priori sample size calculation was performed using G*Power version 3.1.9.7 for a two-sided Wilcoxon–Mann–Whitney test with an α error probability of 0.05, a power of 0.95, and an allocation ratio of 1:1, indicating that a minimum of 31 participants per group (total *n* = 62) was required.

### 2.8. Ethical Approval

The study was approved by the Etlik City Hospital Clinical Research Ethics Committee (Date: 30 October 2024; Decision No.: AEŞH-BADEK-2024-994). Written informed consent was obtained from all participants.

## 3. Results

The study comprised 44 cases diagnosed with fetal growth restriction (FGR) and 43 cases in the control group. Table 1 presents a comparison of the maternal, ultrasonographic, and perinatal outcomes between the two groups.

In the FGR group, maternal age (*p* = 0.022), gravidity (*p* = 0.041), and maternal BMI (*p* = 0.017) were significantly lower than in the control group. ISM1 levels, expressed as median (interquartile range), were significantly lower in the FGR group compared to controls [10.63 (9.95–12.10) vs. 12.40 (10.75–13.25), *p* = 0.003]. The FGR group exhibited substantially decreased gestational age at birth and birth weight (*p* < 0.001 for both). Among Doppler parameters, MCA PI values were significantly lower in the FGR group (*p* = 0.010). The other Doppler parameters and CPR values did not differ significantly between the groups (all *p* > 0.05). The Apgar values at the 1st and 5th minutes were significantly lower in the FGR group (*p* = 0.005 and *p* = 0.002). Fetal distress occurred more frequently in the FGR group (27.3% compared to 2.4%, *p* = 0.001). The prevalence of CAPO (composite adverse perinatal outcome) was 31.8% in the FGR group and 7.0% in the control group (*p* = 0.003).

Among women with fetal growth restriction, cases were further classified according to the presence or absence of composite adverse perinatal outcome (CAPO). Maternal age was significantly lower in the CAPO group (23.7 ± 4.3 years vs. 27.2 ± 4.9 years, *p* = 0.029). No notable variation was observed in the remaining maternal, ultrasonographic, or perinatal characteristics. Likewise, maternal serum ISM1 levels did not differ significantly between women with and without CAPO (*p* = 0.121) (Table 2).

In the correlation analysis performed in the entire cohort (Table 3), Isthmin levels were inversely correlated with the presence of FGR (r = −0.318, *p* = 0.003) and positively correlated with EFW percentile (r = 0.235, *p* = 0.028). No significant correlation was observed between Isthmin levels and maternal BMI in the entire cohort (r = −0.005, *p* = 0.961), the control group (r = −0.091, *p* = 0.560), or the FGR group (r = −0.086, *p* = 0.577). In the FGR group, gestational age at sampling was significantly and inversely correlated with Isthmin levels (r = −0.383, *p* = 0.010).

To address the potential influence of between-group differences and baseline covariates, within-group partial correlation analyses were performed (Table 4). After adjustment for maternal age, BMI, and gestational age at sampling, ISM1 was not significantly correlated with BMI or maternal age in either the control group or the FGR group. In the control group, no significant association was observed between ISM1 and gestational age (partial r = −0.118, *p* = 0.464). In contrast, within the FGR group, ISM1 showed a significant inverse correlation with gestational age after adjustment for maternal age and BMI (partial r = −0.368, *p* = 0.017).

In univariable logistic regression analysis, higher Isthmin levels were significantly associated with lower odds of FGR (β = −0.226, OR = 0.798, 95% CI: 0.655–0.973, *p* = 0.026). Maternal age (β = −0.101, OR = 0.904, 95% CI: 0.829–0.986, *p* = 0.023) and BMI (β = −0.125, OR = 0.883, 95% CI: 0.796–0.979, *p* = 0.019) were also significantly associated with FGR in the univariable analysis, whereas gravidity (β = −0.327, OR = 0.721, 95% CI: 0.496–1.049, *p* = 0.087) was not significantly associated with FGR. In the multivariable Firth penalized logistic regression model, Isthmin remained independently associated with lower odds of FGR (β = −0.224, adjusted OR = 0.805, 95% CI: 0.662–0.979, *p* = 0.030). Maternal age (β = −0.056, adjusted OR = 0.945, 95% CI: 0.857–1.042, *p* = 0.260), BMI (β = −0.098, adjusted OR = 0.906, 95% CI: 0.809–1.015, *p* = 0.088), and gravidity (β = −0.203, adjusted OR = 0.816, 95% CI: 0.549–1.214, *p* = 0.317) were not independently associated with FGR (Table 5).

**Figure 1:**
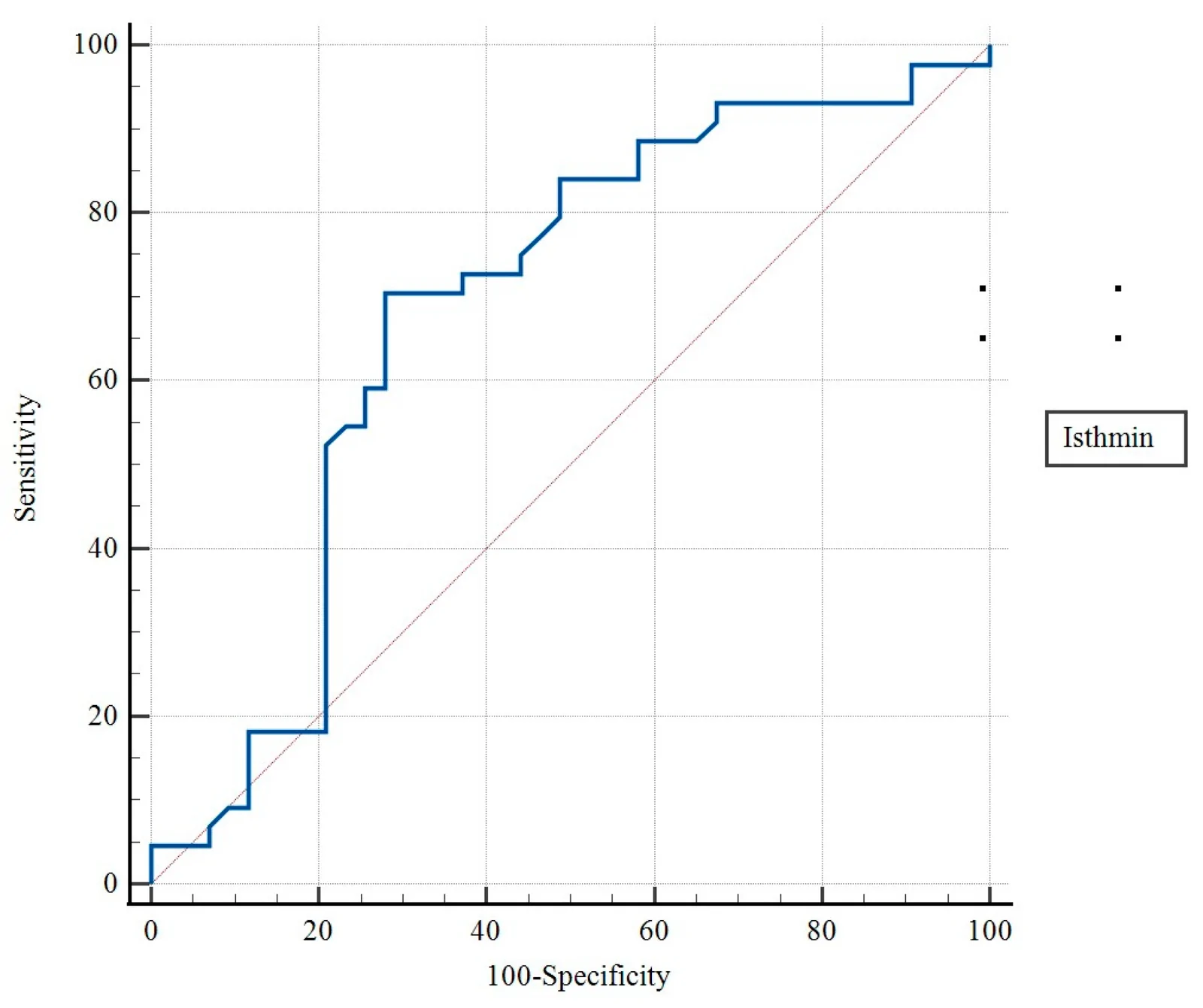
Receiver operating characteristic (ROC) curve of Isthmin levels for the discrimination of fetal growth restriction. The blue line represents the ROC curve for Isthmin, and the diagonal reference line represents the line of no discrimination.

ROC analysis demonstrated that an Isthmin level of ≤11.5 was the optimal cut-off for identifying FGR, with an AUC of 0.684 (95% CI: 0.575–0.779), sensitivity of 70.5% (95% CI: 54.8–83.2), and specificity of 72.1% (95% CI: 56.3–84.7) (Figure 1). The ROC curve demonstrated a moderate discriminative ability, lying consistently above the reference line without approaching the upper left corner, indicating fair but not excellent diagnostic performance.

**Figure 2:**
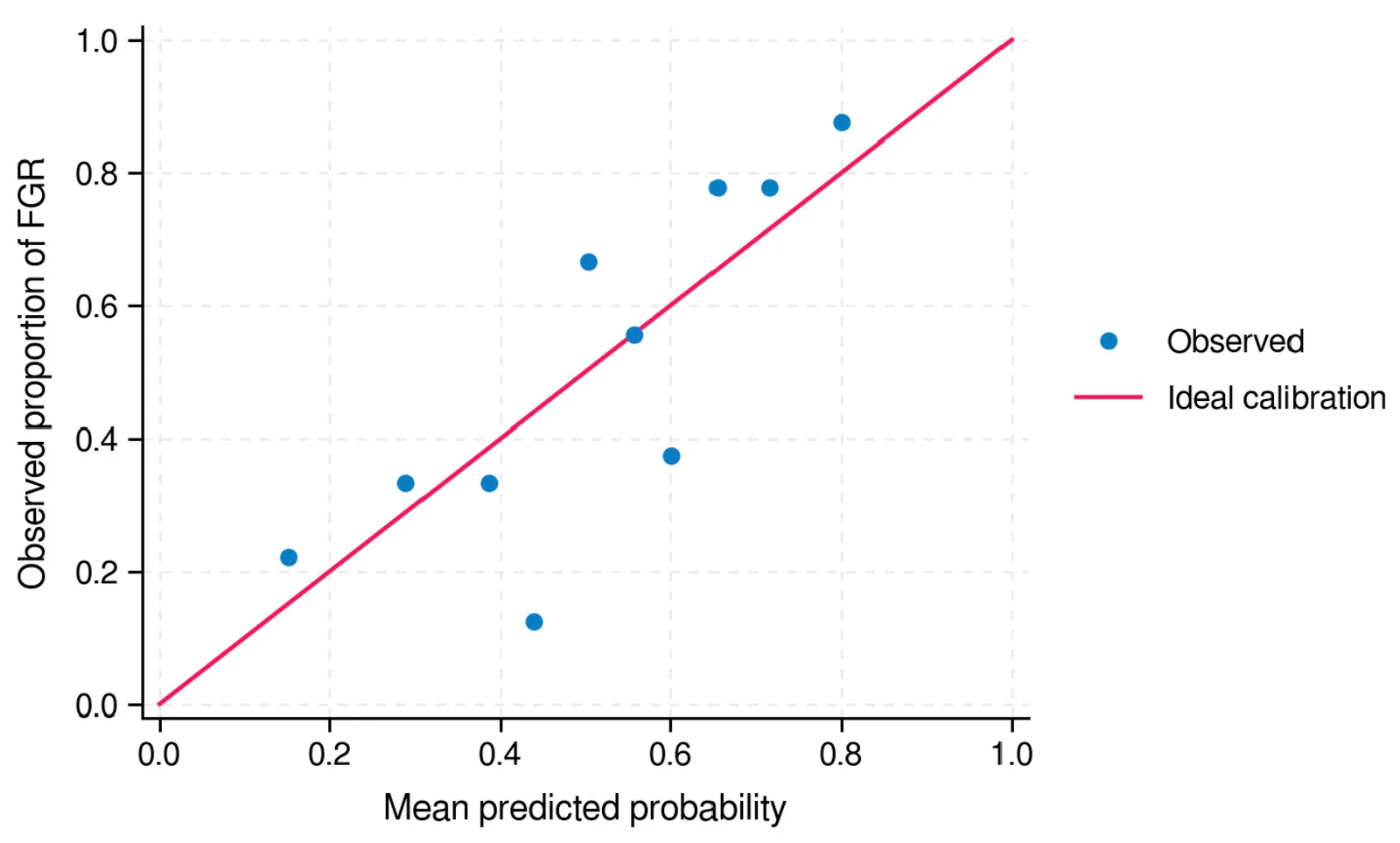
Calibration plot of the multivariable Firth penalized logistic regression model for fetal growth restriction. Observed proportions of fetal growth restriction are plotted against the mean model-estimated probabilities across deciles. The diagonal line represents ideal calibration, whereas the points represent the observed proportions within each group.

The linearity assumption was not violated for Isthmin (*p* = 0.178), maternal age (*p* = 0.286), or BMI (*p* = 0.172). Although the spline-based assessment suggested nonlinearity for gravidity (*p* = 0.038), a quadratic sensitivity analysis did not demonstrate statistically significant nonlinearity (*p* = 0.144); therefore, gravidity was retained as a continuous covariate in the primary model. The multivariable Firth logistic regression model demonstrated an AUC of 0.735 (95% CI, 0.629–0.841). Calibration was good, with a calibration intercept of −0.010 (95% CI, −0.472 to 0.452; *p* = 0.966) and a calibration slope of 1.089 (95% CI, 0.465–1.714; *p* = 0.779) (Figure 2). The Brier score was 0.209.

The corresponding positive and negative likelihood ratios were 2.52 and 0.41, respectively, and the ROC analysis was statistically significant (*p* = 0.002) (Table 6).

## 4. Discussion

The objective of this study was to evaluate maternal serum ISM1 levels in pregnancies complicated by fetal growth restriction (FGR) and to compare them with those of healthy pregnant controls. Maternal serum ISM1 levels were significantly lower in pregnancies affected by FGR than in healthy pregnancies. Furthermore, ISM1 concentrations were negatively associated with the presence of FGR and positively correlated with estimated fetal weight percentiles. However, no association was found between ISM1 levels and composite adverse perinatal outcomes (CAPO). Receiver operating characteristic analysis demonstrated moderate discriminative ability for FGR, with an area under the curve (AUC) of 0.684, a sensitivity of 70.5%, and a specificity of 72.1% at a cut-off value of ≤11.5 ng/mL. These findings suggest an association between lower maternal serum ISM1 levels and impaired fetal growth. However, the moderate diagnostic performance and lack of association with CAPO limit its current clinical applicability as a standalone biomarker. The association between ISM1 and FGR remained significant after adjustment for selected maternal characteristics, supporting further investigation of ISM1 as an exploratory biomarker in larger and externally validated cohorts.

The lower gestational age at delivery observed in the FGR group reflects contemporary obstetric management, in which pregnancies affected by FGR are frequently delivered earlier to mitigate the risk of adverse perinatal outcomes. Similarly, the significantly lower birth weight in the FGR group is an expected finding, reflecting the underlying pathophysiology of impaired fetal growth rather than a novel observation.

These findings should be interpreted in the context of the diverse biological functions of ISM1. Initially described as an angiogenesis inhibitor in experimental tumour models [17], ISM1 has subsequently been implicated in metabolic, developmental, and immune regulatory processes [22,23]. ISM1 has also been identified as an adipokine, and experimental studies have shown that recombinant ISM1 promotes glucose uptake and improves glucose tolerance and hepatic steatosis independently of insulin [18]. In clinical studies, circulating ISM1 levels have been associated with albuminuria in individuals with type 2 diabetes mellitus and with abdominal adipose tissue distribution in individuals with obesity [24,25]. In pregnancy, a case–control study reported higher serum ISM1 concentrations in women with gestational diabetes mellitus than in healthy pregnant controls, suggesting an association between ISM1 and maternal metabolic adaptation during pregnancy [20].

Conversely, the present study demonstrated lower maternal serum ISM1 concentrations in pregnancies affected by FGR, a condition commonly associated with placental insufficiency. This contrasting pattern suggests that ISM1 regulation during pregnancy may be context-dependent. One possible explanation is that maternal metabolic alterations may influence circulating ISM1 differently from the placental dysfunction associated with FGR; however, this hypothesis was not directly investigated in the present study. Experimental evidence supports a role for ISM1 in vascular biology, as ISM1 has been shown to bind integrin αvβ5 and induce endothelial cell apoptosis, thereby exerting anti-angiogenic effects [17]. Whether alterations in this pathway influence placental vascular development in FGR remains unknown and requires direct investigation. ISM1 has also been shown to modulate NODAL signalling, a pathway involved in developmental patterning and organ morphogenesis [19]. Although these findings provide a biological basis for further investigation, the potential involvement of ISM1-related developmental signalling in placental development and fetal growth remains hypothetical. Therefore, the lower maternal serum ISM1 concentrations observed in our FGR cohort should be interpreted as an association rather than evidence of a direct mechanistic role in placental dysfunction.

Given the limited obstetric literature on the Isthmin protein family, findings concerning the related protein Isthmin-2 (ISM2) may provide relevant context. ISM2 is expressed in placental tissue and has been reported to be decreased in preeclampsia [21]. Since abnormal placental angiogenesis is an important feature of both preeclampsia and FGR, the reduction in maternal serum ISM1 observed in our FGR cohort may suggest that members of the Isthmin family warrant further investigation in placental insufficiency disorders. However, the biological functions of ISM1 and ISM2 should not be assumed to be equivalent, and our findings do not establish a common mechanistic pathway between these conditions.

Current clinical guidelines emphasize ultrasound assessment, Doppler velocimetry, and maternal risk factor stratification in the evaluation and management of pregnancies affected by FGR [3,6,26]. Despite advances in fetal surveillance, there remains an interest in identifying biomarkers that could complement established clinical and ultrasonographic parameters. Previous studies have evaluated several circulating biomarkers, including angiogenic factors, for the identification of FGR; however, their overall diagnostic performance has generally been insufficient to support routine clinical use in isolation [27]. In the present study, ISM1 demonstrated moderate discriminative ability for FGR. Importantly, we did not directly measure PlGF, sFlt-1, or the sFlt-1/PlGF ratio; therefore, no conclusions can be drawn regarding whether ISM1 provides superior or additional diagnostic value compared with these established angiogenic biomarkers. Further studies directly comparing ISM1 with established biomarkers and evaluating their combined performance are required before any incremental diagnostic value can be established. The investigation of emerging biomarkers may ultimately contribute to efforts to distinguish pathological fetal growth restriction from constitutionally small but healthy fetuses [28].

Although maternal serum ISM1 levels were significantly lower in pregnancies affected by fetal growth restriction (FGR), ISM1 was not associated with adverse perinatal outcomes in our cohort. This suggests that the observed association between ISM1 and FGR does not necessarily extend to predicting neonatal morbidity. As perinatal outcomes are influenced by multiple factors, including gestational age at delivery, obstetric complications and neonatal clinical conditions, the prognostic performance of a single circulating biomarker may be limited. Therefore, the present findings do not support the use of ISM1 as an independent predictor of adverse perinatal outcomes. Future studies should evaluate whether combining ISM1 with established Doppler indices or angiogenic biomarkers provides additional prognostic information beyond that provided by existing clinical parameters.

There are several hypotheses that could explain the observed association between lower maternal serum ISM1 levels and FGR. ISM1 levels could potentially be associated with changes in placental angiogenesis, maternal–foetal nutrient transfer, metabolic adaptation or developmental signalling. However, none of these mechanisms were investigated directly in the present study. Therefore, these interpretations are speculative and do not constitute evidence of a causal role for ISM1 in the pathogenesis of FGR. Further research incorporating placental tissue analysis and appropriate mechanistic models is required to determine whether altered ISM1 levels are directly involved in the pathophysiology of FGR or if they represent a circulating marker associated with impaired fetal growth.

This study has several strengths, including its prospective design, standardised ultrasonographic and Doppler assessments, and evaluation of the independent association between maternal serum ISM1 levels and fetal growth restriction (FGR) after adjustment for selected maternal characteristics. However, several limitations should be acknowledged. Firstly, this was a single-centre study with a relatively small sample size, which may limit the generalisability of the findings. In addition, the inclusion of only healthy pregnancies as a comparator group does not allow us to determine whether reduced maternal serum ISM1 concentrations are specific to FGR or represent a more general association with placental dysfunction or other pregnancy complications. The absence of disease comparator groups, such as preeclampsia or gestational hypertension without FGR, gestational diabetes, and constitutionally small-for-gestational-age pregnancies, therefore limits conclusions regarding the disease specificity of ISM1. Secondly, the proposed ISM1 cut-off value was derived from the same cohort in which its diagnostic performance was evaluated and has not been validated externally; therefore, this threshold should be considered preliminary. Thirdly, ISM1 was measured at a single time point, which precluded the assessment of longitudinal changes in maternal serum concentrations throughout pregnancy. Fourthly, placental tissue expression of ISM1 was not evaluated, which limits our ability to draw conclusions about the relationship between circulating ISM1 and placental function. Finally, although the multivariable analysis was adjusted for maternal age, BMI and number of pregnancies, residual metabolic confounding cannot be ruled out. Pre-pregnancy or booking BMI, gestational weight gain, and systematic insulin and lipid measurements were not available for analysis, which further limits a comprehensive assessment of potential metabolic confounding. This is a particularly relevant limitation because BMI was significantly higher in the control group, and because ISM1 has been characterised as an adipokine. Accordingly, the independent association between ISM1 and FGR observed after statistical adjustment should be interpreted with caution.

Our findings have the potential to inform future research rather than immediate clinical application. The observed reduction in maternal serum ISM1 levels in FGR supports its further evaluation as a candidate exploratory biomarker. However, given its moderate standalone diagnostic performance, the present findings do not support the use of ISM1 as an independent clinical diagnostic marker. Future studies should determine whether ISM1 provides additional diagnostic information when combined with established ultrasound, Doppler, and angiogenic markers. Further research is required before the potential clinical utility of ISM1 can be established, including larger multicentre cohorts, external validation of the proposed cut-off value, longitudinal assessment of ISM1 across gestation, and mechanistic studies examining placental expression and function.

In summary, maternal serum ISM1 levels were significantly lower in pregnancies affected by FGR, suggesting a potential association between ISM1 and impaired fetal growth. However, its moderate diagnostic performance and lack of association with adverse perinatal outcomes limit its current applicability as a standalone diagnostic or prognostic marker. Therefore, ISM1 should currently be considered a promising exploratory biomarker rather than a marker suitable for clinical implementation. Larger multicentre studies with external validation are required to confirm these findings and to determine whether ISM1 provides additional diagnostic value when incorporated into multimarker models alongside Doppler indices and established angiogenic biomarkers.

## Figures and Tables

**Table 1:** Comparison of maternal, ultrasonographic, and perinatal outcomes between FGR and control groups.

Variables	FGR (*n* = 44)	Control (*n* = 43)	*p*-Value
**Maternal Age (years)**	26 (23–29)	28 (25–31)	0.022 ^a^
**Gestational Age at Sampling (weeks)**	37 (36–38)	36 (35–37)	0.114 ^a^
**Gravidity**	1 (1–3)	2 (1–3)	0.041 ^a^
**Parity**	0 (0–1)	1 (0–1)	0.066 ^a^
**BMI (kg/m^2^)**	28.87 (25.34–32.25)	31.14 (28.40–33.06)	0.017 ^a^
**ISM1 (ng/mL)**	10.63 (9.95–12.10)	12.40 (10.75–13.25)	0.003 ^a^
**Ultrasonographic Measurements**			
**AC (mm)**	296 ± 20	320 ± 12	<0.001 ^b^
**AC Percentile (%)**	1.0 (1.0–3.0)	50.0 (30.0–62.0)	<0.001 ^a^
**EFW (gr)**	2370 ± 356	2837 ± 311	<0.001 ^b^
**EFW Percentile (%)**	3.5 (2.0–5.0)	43.0 (30.0–63.0)	<0.001 ^a^
**MVP (mm)**	43 (39–50)	48 (40–55)	0.142 ^a^
**UA S/D**	2.47 ± 0.51	2.57 ± 0.48	0.348 ^b^
**UA PI**	0.90 (0.80–0.95)	0.91 (0.77–1.04)	0.505 ^a^
**MCA S/D**	3.70 (3.30–4.00)	3.70 (3.40–3.83)	0.993 ^a^
**MCA PI**	1.25 (1.15–1.35)	1.32 (1.27–1.37)	0.010 ^a^
**CPR**	1.35 ± 0.32	1.44 ± 0.34	0.178 ^b^
**Perinatal Outcomes**			
**Gestational Age at Birth (weeks)**	37.2 (37.0–38.3)	39.2 (38.2–40.5)	<0.001 ^a^
**Birth Weight (g)**	2497 ± 338	3291 ± 448	<0.001 ^b^
**Apgar score at 1st minute**	8 (7–9)	9 (8–9)	0.005 ^a^
**Apgar score at 5th minute**	9 (9–10)	10 (9–10)	0.002 ^a^
**5th minute Apgar score ≤7**	4 (9.1%)	-	0.116 ^c^
**Fetal Distress**	12 (27.3%)	1 (2.4%)	0.001 ^c^
**NICU Admission**	4 (9.1%)	2 (4.7%)	0.676 ^c^
**CPAP Requirement**	2 (4.5%)	-	0.494 ^c^
**Requirement for Mechanical Ventilation**	1 (2.3%)	-	>0.99 ^c^
**CAPO**	14 (31.8%)	3 (7.0%)	0.003 ^c^

^a^ The Mann–Whitney U test was used for comparisons between groups. Data are presented as median (interquartile range). ^b^ Independent samples *t*-test was used for comparisons between groups. Data are presented as mean ± standard deviation. ^c^ Categorical variables were compared using the chi-square or Fisher’s exact test, as appropriate. Results are shown as *n* (%). Abbreviations: FGR, Fetal Growth Restriction; BMI, Body Mass Index; AC, abdominal circumference; EFW, estimated fetal weight; MVP, maximum vertical pocket; UA, Umbilical artery; S/D, systolic/diastolic ratio; PI, pulsatility index; MCA, Middle cerebral artery; CPR, Cerebroplacental ratio; NICU, neonatal intensive care unit; CPAP, continuous positive airway pressure; CAPO, composite adverse perinatal outcome. Composite adverse perinatal outcome (CAPO) was defined as the presence of at least one of the following: 5-min Apgar score ≤ 7, fetal distress, NICU admission, requirement for CPAP, or mechanical ventilation.

**Table 2:** Comparison of maternal, ultrasonographic, and perinatal characteristics between FGR cases with and without CAPO.

Variables	CAPO (*n* = 14)	Non-CAPO (*n* = 30)	*p*-Value
**Maternal Age (years)**	23.7 ± 4.3	27.2 ± 4.9	0.029 ^a^
**BMI (kg/m^2^)**	28.61 (24.91–32.85)	28.87 (25.48–31.65)	0.960 ^b^
**Gestational Age at Sampling (weeks)**	36.4 ± 2.1	36.9 ± 1.7	0.486 ^a^
**ISM1 (ng/mL)**	11.15 (10.25–12.85)	10.43 (9.65–11.50)	0.121 ^b^
**Gestational Age at Birth (weeks)**	37.1 ± 2.0	37.5 ± 1.1	0.311 ^a^
**Birth Weight (g)**	2456 ± 377	2516 ± 323	0.593 ^a^
**Ultrasonographic Measurements**			
**AC (mm)**	295 ± 20	297 ± 20	0.804 ^a^
**AC Percentile (%)**	1.0 (1.0–3.0)	1.0 (1.0–3.0)	0.966 ^b^
**EFW (gr)**	2490 (2180–2564)	2398 (2270–2590)	0.950 ^b^
**EFW Percentile (%)**	3.8 ± 2.6	3.9 ± 2.4	0.885 ^a^
**MVP (mm)**	43 ± 12	44 ± 10	0.699 ^a^
**UA S/D**	2.62 ± 0.50	2.40 ± 0.51	0.185 ^a^
**UA PI**	0.90 ± 0.19	0.90 ± 0.17	0.989 ^a^
**MCA S/D**	3.56 (3.30–4.00)	3.75 (3.30–4.00)	0.419 ^b^
**MCA PI**	1.24 (1.15–1.30)	1.27 (1.15–1.35)	0.527 ^b^
**CPR**	1.29 ± 0.33	1.37 ± 0.32	0.466 ^a^

^a^ Independent samples *t*-test was used for comparisons between groups. Data are presented as mean ± standard deviation. ^b^ The Mann–Whitney U test was used for comparisons between groups. Data are presented as median (interquartile range). Abbreviations: CAPO, composite adverse perinatal outcome; FGR, fetal growth restriction; BMI, body mass index; AC, abdominal circumference; EFW, estimated fetal weight; MVP, maximum vertical pocket; UA, umbilical artery; S/D, systolic/diastolic ratio; PI, pulsatility index; MCA, middle cerebral artery; CPR, cerebroplacental ratio.

**Table 3:** Correlation analysis of Isthmin levels with maternal, ultrasonographic, and perinatal variables.

Variables	r	*p*-Value
**Analysis performed in the entire cohort**		
**BMI (kg/m^2^)**	−0.005	0.961 ^b^
**FGR**	−0.318	0.003 ^a^
**AC Percentile (%)**	0.189	0.079 ^b^
**EFW Percentile (%)**	0.235	0.028 ^b^
**Analysis performed only in the control cohort**		
**Gestational Age at Sampling (weeks)**	−0.089	0.568 ^b^
**Maternal Age (years)**	−0.091	0.560 ^b^
**BMI (kg/m^2^)**	−0.091	0.560 ^b^
**AC Percentile (%)**	−0.286	0.063 ^b^
**EFW Percentile (%)**	−0.197	0.206 ^b^
**MVP (mm)**	0.137	0.380 ^b^
**UA S/D**	−0.096	0.540 ^b^
**UA PI**	−0.125	0.425 ^b^
**MCA S/D**	0.010	0.950 ^b^
**MCA PI**	0.169	0.279 ^b^
**CPR**	0.163	0.297 ^b^
**Analysis performed only in the FGR cohort**		
**Gestational Age at Sampling (weeks)**	−0.383	0.010 ^b^
**Maternal Age (years)**	−0.018	0.909 ^b^
**BMI (kg/m^2^)**	−0.086	0.577 ^b^
**AC Percentile (%)**	−0.125	0.420 ^b^
**EFW Percentile (%)**	0.027	0.861 ^b^
**MVP (mm)**	0.121	0.433 ^b^
**UA S/D**	0.135	0.384 ^b^
**UA PI**	0.170	0.271 ^b^
**MCA S/D**	0.122	0.432 ^b^
**MCA PI**	0.068	0.660 ^b^
**CPR**	−0.173	0.260 ^b^

^a^ Point-biserial correlation was used for analyses between categorical and continuous variables. ^b^ Spearman’s rank correlation was used for analyses between continuous variables. Abbreviations: FGR, fetal growth restriction; AC, abdominal circumference; EFW, estimated fetal weight; BMI, body mass index; MVP, maximum vertical pocket; UA, umbilical artery; S/D, systolic/diastolic ratio; PI, pulsatility index; MCA, middle cerebral artery; CPR, cerebroplacental ratio.

**Table 4:** Within-group partial correlation analysis of Isthmin levels adjusted for maternal BMI, maternal age, and gestational age.

Variable	Control Group (*n* = 43)		FGR Group *(n* = 44)	
	Partial r	*p*-Value	Partial r	*p*-Value
EFW percentile (%)	−0.215	0.177	0.031	0.848
BMI (kg/m^2^)	−0.064	0.692	−0.018	0.913
Maternal age (years)	−0.111	0.488	0.020	0.900
Gestational age at sampling (weeks)	−0.118	0.464	−0.368	0.017

Note: Partial correlation coefficients were calculated separately within the control and FGR groups, with adjustment for maternal BMI, maternal age, and gestational age at sampling. For each variable, the correlation represents its association with Isthmin levels independent of the other covariates.

**Table 5:** Univariable logistic regression and multivariable Firth penalized logistic regression analysis for fetal growth restriction.

Variable	Univariable				Multivariable			
	β (Coefficient)	OR	95% CI	*p*-Value	β (Coefficient)	aOR	95% CI	*p*-Value
**Isthmin**	−0.226	0.798	0.655–0.973	0.026	−0.224	0.805	0.662–0.979	0.030
**Maternal Age, years**	−0.101	0.904	0.829–0.986	0.023	−0.056	0.945	0.857–1.042	0.260
**BMI (kg/m^2^)**	−0.125	0.883	0.796–0.979	0.019	−0.098	0.906	0.809–1.015	0.088
**Gravidity**	−0.327	0.721	0.496–1.049	0.087	−0.203	0.816	0.549–1.214	0.317

**Table 6:** Discriminative performance of maternal serum ISM1 for fetal growth restriction.

Variable	Cut-Off	AUC (95% CI)	Sensitivity (95% CI)	Specificity (95% CI)	LR^+^	LR^−^	*p*-Value
**Isthmin (ng/mL)**	≤11.5	0.684 (0.575–0.779)	70.5% (54.8–83.2)	72.1% (56.3–84.7)	2.52	0.41	0.002

Abbreviations: AUC, area under the curve; CI, confidence interval; LR^+^, positive likelihood ratio; LR^−^, negative likelihood ratio.

## Data Availability

The data presented in this study are available from the corresponding author upon reasonable request.

## Abbreviations

The following abbreviations are used in this manuscript:

ISM1	Isthmin-1
FGR	Fetal Growth Restriction
EFW	Estimated Fetal Weight
UA-PI	Umbilical Artery Pulsatility Index
MCA-PI	Middle Cerebral Artery Pulsatility Index
CPR	Cerebroplacental Ratio
ELISA	Enzyme-Linked Immunosorbent Assay
ROC	Receiver Operating Characteristic
AUC	Area Under the Curve
AC	Abdominal Circumference
FIGO	International Federation of Gynecology and Obstetrics
ACOG	American College of Obstetricians and Gynecologists
ISUOG	International Society of Ultrasound in Obstetrics and Gynecology
RCOG	Royal College of Obstetricians and Gynaecologists
CVD	Cardiovascular Disease
T2DM	Type 2 Diabetes Mellitus
MetS	Metabolic Syndrome
PlGF	Placental Growth Factor
sFlt-1	Soluble Fms-like Tyrosine Kinase-1
TSR1	Thrombospondin Type 1 Repeat
AMOP	Adhesion-associated domain in MUC4 and Other Proteins
GRP78	Glucose-Regulated Protein 78
VEGF	Vascular Endothelial Growth Factor
bFGF	Basic Fibroblast Growth Factor
AKT	Protein Kinase B (PKB)
CAPO	Composite Adverse Perinatal Outcome
MVP	Maximum Vertical Pocket
S/D	Systolic/Diastolic Ratio
aORs	Adjusted Odds Ratios
CIs	Confidence Intervals
BMI	Body Mass Index
NICU	Neonatal Intensive Care Unit
GDM	Gestational Diabetes Mellitus
